# Incidental renal pelvis and ureteral verrucous carcinoma: A case report and literature review

**DOI:** 10.1097/MD.0000000000047972

**Published:** 2026-03-06

**Authors:** Zhiyun Yang, Yidao Liu, Xingming Zhang, Lingying Song, Jiafu Jiang, Jun Pan, Jiasheng Li, Ji Li

**Affiliations:** a Department of Urology, Dehong Prefecture People’s Hospital, Dehong, China; b Department of Urology, West China Hospital, Sichuan University, Chendu, China; c Department of Pathology, Dehong Prefecture People’s Hospital, Dehong, China; d Department of Radiology, Dehong Prefecture People’s Hospital, Dehong, China.

**Keywords:** renal pelvis, squamous cell carcinoma, ureter, verrucous carcinoma

## Abstract

**Rationale:**

Verrucous carcinoma (VC) of the upper urinary tract is an extremely rare disease, with only 7 cases reported in the literature to date. We report a successfully treated case of VC involving the renal pelvis and ureter, along with a literature review to explore its pathogenesis and optimal treatment strategies.

**Patient concerns:**

A 79-year-old male presented with “recurrent right flank pain for over 10 years and fever for 1 week.”

**Diagnoses:**

VC of the right renal pelvis and ureter (pT1N0M0); right renal and ureteral calculi and right pyonephrosis.

**Interventions:**

After initial percutaneous nephrostomy to manage the infection, the patient underwent nephrectomy and partial ureterectomy.

**Outcomes:**

Histopathological analysis revealed VC in the renal pelvis and ureter, with no muscular invasion or lymph node metastasis. Postoperative recovery was uneventful, and at the 3-month follow-up, no recurrence or residual disease was observed on imaging and cystoscopy.

**Lessons:**

patients with long-standing renal calculi and chronic inflammation should be carefully monitored for potential malignant transformation. Early treatment of predisposing factors is essential. Contrast-enhanced imaging and thorough pathological evaluation can aid in diagnosis.

## 1. Introduction

Verrucous carcinoma (VC) is a rare, well-differentiated variant of squamous cell carcinoma (SCC) characterized by low metastatic potential and indolent clinical behavior. VC most commonly arises in the oral cavity, larynx, esophagus, nasal cavity, vulva, penis, anorectal region, and skin.^[1]^ Its occurrence in the upper urinary tract is exceedingly rare, with only 7 cases reported in the literature. Here, we report a unique case of VC involving both the renal pelvis and ureter in a 79-year-old male, the oldest patient documented to date with synchronous involvement of both anatomical sites. This report also includes a review of the relevant literature to contextualize the clinical and pathological features of this unusual malignancy.

## 2. Case report

**Figure 1.**
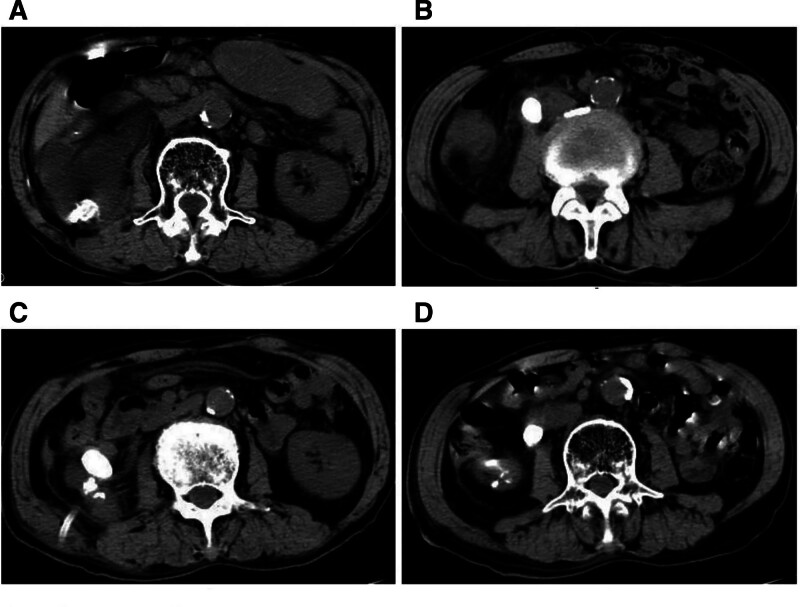
Preoperative imaging findings for nephrectomy. (A and B) CT imaging demonstrated right renal and proximal ureteral calculi with severe hydronephrosis; (C and D) Post-right nephrostomy CT scan showed atrophy of the right kidney with significant improvement in right hydronephrosis. CT = computed tomography.

A 79-year-old male was referred to our hospital on January 8th, 2025, due to recurrent right flank pain for over 10 years and fever for 1 week. The patient has a 16-year history of right kidney stones. Sixteen years ago, due to right-sided lower back pain, the patient was diagnosed at a local hospital with multiple right kidney stones (0.5–1.2cm) and a right ureteral stone (0.6 cm), accompanied by mild right kidney hydronephrosis. The patient underwent 2 weeks of oral medication for stone expulsion and spontaneously passed 1 stone, with symptom relief. Over the past decade, the patient has intermittently experienced dull pain in the right side of back but did not seek further medical attention. Additionally, The patient has a 40-year smoking history with heavy daily consumption but has been smoke-free for 6 years. His past medical history included hypertension, splenectomy, without family history of malignancy. This patient received a 1-week course of ceftazidime for anti-infective treatment outside the hospital, but his symptoms showed no improvement. Physical examination revealed right upper abdominal tenderness and right renal percussion pain. Urine culture identified Enterococcus faecium infection. Non-enhanced computed tomography (CT) revealed right renal and proximal ureteral calculi with the largest stone measuring 2.6 cm in diameter, and severe right kidney hydronephrosis. (Fig. 1A and B). Despite treatment with amikacin(0.2 g was administered intravenously every 12 hours) for 1 week (No adverse drug reactions), the patient’s symptoms persisted. After obtaining the patient’s consent, further percutaneous nephrostomy was performed on January 15th, 2025, draining copious purulent fluid (culture-positive for Enterococcus faecium). Tuberculosis tests were negative. The patient’s symptoms improved after drainage, with a nephrostomy output of <50 ml per day. Follow-up CT showed reduced hydronephrosis and right kidney atrophy (Fig. 1C and D).

After infection control, we recommended right nephrectomy for definitive infection management. The patient consented to the treatment plan and underwent laparoscopic right nephrectomy on January 24th, 2025. The surgical specimen included the kidney, perirenal fat, and proximal ureter above the pelvic brim. Despite severe perirenal and periureteral adhesions due to chronic infection, the surgery was uneventful with no intraoperative complications occurred. Postoperative stone composition analysis indicated calcium oxalate monohydrate stones. The patient recovered well postoperatively, with no fever recurrence. He was highly satisfied with the treatment outcome. Drains were removed on postoperative day 3th, and the patient was discharged on day 5th.

**Figure 2.**
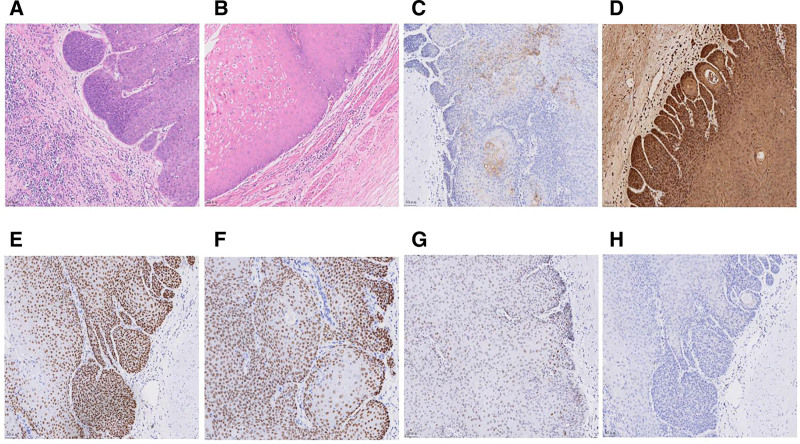
HE staining and IHC staining of the resected specimen. (A) HE staining of renal pelvis VC (X200); (B) HE staining of ureteral VC (X200); (C) IHC staining shows a focal positive expression of CK7(X200); (D) IHC staining shows a strongly positive expression of CK5/6 (200X); (E) IHC staining shows a positive expression of P63(X200); (F) IHC staining shows a positive expression of P40(X200); (G) IHC staining shows a positive expression of P53(X200); (H) IHC staining shows a negative expression of P16(X200). HE = hematoxylin-eosin, IHC = Immunohistochemistry, VC = verrucous carcinoma.

Histopathological analysis revealed VC in the renal pelvis (2.5 × 1.8 × 0.7 cm) and ureter (1.5 × 1.2 × 0.5 cm), confined to the lamina propria without muscular invasion. Surgical margins were negative, and no lymph node metastasis was found. Additional findings included chronic pyelonephritis, ureteral inflammation, squamous metaplasia, and renal/ureteral calculi. Immunohistochemistry (IHC) showed CK7 (focal +), CK5/6 (++), P63 (+), P40 (+), P53 (+), P16 (−), Ki-67 (15% positivity), GATA-3 (−), UPK-2 (−) and CK20 (−) (Fig. 2). Final pathological diagnosis: VC of the renal pelvis and ureter (pT1N0M0). Due to this incidental finding, we recommended further surgical resection of the residual ureter. The patient declined additional surgery and opted for close follow-up.

**Figure 3.**
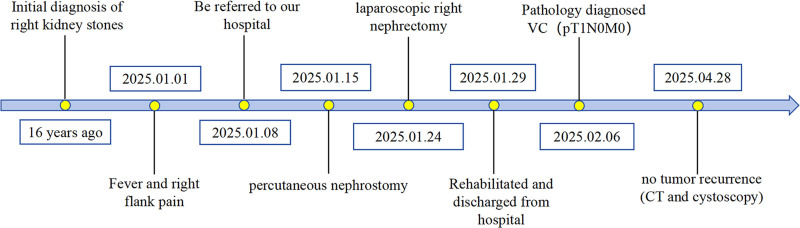
The timeline of our patient’s treatment process.

At the 3-month follow-up, contrast-enhanced CT and cystoscopy revealed no tumor recurrence in the ureteral stump or bladder. The patient remains under regular surveillance. The patient’s diagnostic and treatment process is detailed in Figure 3.

## 3. Discussion

VC is an exceptionally rare subtype of SCC, first described by Ackerman in 1948.^[2]^ It is typified by exophytic, wart-like growths, a pushing (non-infiltrative) margin, and minimal cytological atypia.^[3]^ While urothelial carcinoma dominates upper urinary tract malignancies, SCC accounts for approximately 1.35%,^[4]^ and VC being exceedingly rare, with just 7 reported cases.^[1,5–9]^ Affected patients (aged 41–79, mean 59) were predominantly male, and all previously reported cases occurred in patients with long-standing renal calculi and chronic infection; Only 1 case involved a horseshoe kidney; None had human papillomavirus (HPV) involvement (Table 1).

**Table 1:** Characteristics of patients with VC of upper urinary tract.

	Age	Sex	Anomaly in tract urinary	Kidney stone	Papillomavirus	Location of lesion	Treatment received	Outcome
Sheaff et al, 1996^[5]^	41	M	+ (horseshoe kidney)	+	−	Renal pelvis	Heminephrectomy	6 mo, NED
Sellami- Boudawara et al, 2001^[6]^	61	M	−	+	Not mentioned	Renal pelvis	Radical nephrectomy	5 yr, NED
Sellami- Boudawara et al, 2001^[6]^	56	M	−	+	Not mentioned	Renal pelvis	Nephroureterectomy	Died
Kansal et al, 2001^[7]^	64	F	−	+	−	Renal pelvis	Radical nephrectomy	2 yr, NED
Basekioglu et al, 2012^[1]^	56	M	−	+	−	Renal pelvis	Radical nephrectomy combined with RT and chemotherapy	3 yr, died
Hung WH et al, 2015^[8]^	53	M	−	+	Not mentioned	Renal pelvis and ureter	Nephroureterectomy combined with RT	3 mo, NED
Ibañez et al, 2023^[9]^	60	M	−	+	Not mentioned	Calyceal diverticulum	Partial nephrectomy	5 yr, NED
Present case	79	M	−	+	Did not check	Renal pelvis and ureter	Nephrectomy and partial ureterectomy	3 mo, NED

“−” = negative, “+” = positive, F = female, M = male, NED = no evidence of disease, RT = radiotherapy, VC = verrucous carcinoma.

The first renal pelvic VC case was reported by Sheaff in 1996, involving a horseshoe kidney with staghorn calculus and chronic pyelonephritis. Isotope scans showed loss of function in the right kidney, and pathological examination after nephrectomy revealed renal pelvic VC. No evidence of HPV infection was found, and author suggested that the VC likely arose from chronic inflammatory stimulation caused by the calculus.^[5]^ All 7 patients of upper urinary tract VC reported in the literature had a long history of renal calculi and chronic infection, with no evidence of papillomavirus infection. These findings suggest chronic irritation and inflammation as likely contributing factors. Similarly, compared to the general population, exposure to cigarette smoke (CS) increases the risk of upper tract urothelial carcinoma by 3 to 7 times.^[10]^ Another study across 3 regions in the United States found that smoking is associated with a 3.1-fold increased risk of renal pelvis and ureteral cancer, with long-term smokers (over 45 years) facing a 7.2-fold increase. Approximately 7 out of ten cases of renal pelvis and ureteral cancer in men and nearly 4 out of ten in women are attributed to smoking.^[11]^ The initial event of CS exposure involves oxidative damage and apoptosis, followed by sustained signaling through epidermal growth factor receptor and mitogen activated protein kinase pathways. CS exposure also leads to hyperphosphorylation of phosphorylated Retinoblastoma protein, activation of cyclin E, and dysregulation of the cell cycle, resulting in infiltration of the renal pelvis lamina propria epithelial cells and the development of pT1 tumors.^[12]^ In this case, the patient had both chronic calculi and a significant smoking history, further supporting these associations.

According to European Association of Urology guidelines,^[10]^ kidney-sparing surgery should be the first-line treatment for low-risk upper tract urothelial carcinoma, as it reduces complications associated with radical surgery (such as loss of renal function) without compromising oncological outcomes. For high-risk localized disease, radical nephroureterectomy (RNU) is the preferred option. Histological subtypes such as micropapillary carcinoma, SCC, and sarcoma are often associated with locally advanced disease and poorer outcomes, and patients with these subtypes should consider RNU after careful discussion. For metastatic upper tract tumors, platinum-based chemotherapy has been the first-line treatment for years, despite limitations in safety and low response rates.^[13]^ For metastatic urothelial carcinoma, pembrolizumab is currently considered as the first-line treatment for patients ineligible for platinum-based therapy and as second-line treatment after platinum chemotherapy.^[14–17]^ Alessandro study suggests that pembrolizumab is effective for patients who previously treated with platinum-based regimens, regardless of sensitivity to first-line treatment or histological type. However, patients with liver metastases and poor Eastern Cooperative Oncology Group performance status derive limited benefit from pembrolizumab.^[18]^ Additionally, recent studies show that the combination of enfortumab vedotin and pembrolizumab offers new hope for treating metastatic urothelial carcinoma.^[10,19]^

Primary SCC of the renal pelvis and ureter typically has a short clinical course, is prone to infiltration and metastasis, and generally has a poor prognosis.^[20]^ Unlike conventional SCC, which typically shows aggressive behavior, VC exhibits slow progression, low metastatic potential, favorable prognosis when completely excised, and surgical resection usually achieves excellent therapeutic outcomes. Jo study on penile VC showed that local excision for tumors <3cm and at pT1 stage resulted in favorable clinical outcomes with rare recurrence.^[21]^ Follow-up data from the 7 reported cases of upper urinary tract VC revealed that 1 patient who received adjuvant radiotherapy and chemotherapy (5-fluorouracil + cisplatin) died 3 years later due to recurrence and liver metastasis. This patient’s pathology was not pure VC but rather a mixed poorly differentiated SCC, and the surgical margin was positive.^[1]^ Another patient died of renal failure without recurrence or metastasis,^[6]^ while the 5 cases of pure VC showed good survival with no evidence of disease at follow-up (3 months to 5 years). Therefore, we suggest that for isolated low-risk localized upper tract VC, kidney-sparing surgery may be the first-line treatment. Depending on tumor characteristics, endoscopic management or segmental ureterectomy with adequate margins can be decided jointly with the patient. For high-risk locally advanced disease, loss of renal function on the affected side, or pyonephrosis, RNU is recommended. For metastatic VC, systemic therapy with platinum-based chemotherapy or immunotherapy is indicated.

In our case, the patient was incidentally diagnosed with VC in both the renal pelvis and ureter postoperatively, with no SCC component. The pathological stage was T1, and margins were negative. Follow-up CT and cystoscopy at 3 months showed no recurrence or metastasis. However, since the patient had undergone preoperative percutaneous nephrostomy and declined further resection of the residual ureter, close surveillance of the residual ureter and surgical site is necessary to monitor for possible recurrence. The recommended follow-up plan includes: cystoscopy and urine cytology every 6 months for 2 years, then annually until 5 years; contrast-enhanced abdominal CT and chest CT every 6 months for 2 years, then annually.

## 4. Conclusion

Patients with long-standing renal calculi and chronic inflammation should be carefully monitored for potential malignant transformation. Early treatment of predisposing factors is essential. Contrast-enhanced imaging and thorough pathological evaluation can aid in diagnosis. The prognosis of VC with a SCC component is worse than that of pure VC, necessitating careful pathological differentiation. Surgical resection is the primary treatment for pure VC and generally leads to excellent outcomes. Vigilant postoperative follow-up is crucial due to the possibility of recurrence. More studies are needed to better understand the pathogenesis, diagnosis, and management of this rare entity.

## Author contributions

**Data curation:** Jiasheng Li.

**Writing – original draft:** Zhiyun Yang, Ji Li.

**Writing – review & editing:** Zhiyun Yang, Yidao Liu, Xingming Zhang, Lingying Song, Jiafu Jiang, Jun Pan, Jiasheng Li, Ji Li.
